# Farber's Lipogranulomatosis: Multimodal Therapy With Tocilizumab and Consolidative HSCT Improves Assessment, and Long‐Term Outcome

**DOI:** 10.1002/jmd2.70041

**Published:** 2025-08-07

**Authors:** Nathanael C. C. Lucas, Claire Horgan, Omima Mustafa, Srividhya Senthil, Denise Bonney, Ramya Nataraj, Sophie Fisher, Chern Tan, Stewart Rust, Simon A. Jones, Sarah Hulley, Robert Wynn

**Affiliations:** ^1^ Paediatric Bone Marrow Transplant Unit, Royal Manchester Children's Hospital Manchester University NHS Trust Manchester UK; ^2^ Willink Unit, St Mary's Hospital Manchester University NHS Trust Manchester UK; ^3^ Paediatric Neuropsychology, Royal Manchester Children's Hospital Manchester UK

**Keywords:** Farber's lipogranulomatosis, haematopoietic stem cell transplant, subcutaneous nodules, tocilizumab

## Abstract

Farber's lipogranulomatosis (FL) is an autosomal recessive lipid storage disorder, arising as a consequence of genetic acid ceramidase deficiency. Clinically, it presents as severe arthritis, voice hoarseness, and widespread, painful subcutaneous nodules (SCN). For those without CNS involvement, haematopoietic stem cell transplant provides a viable option for the improvement of both respiratory and musculoskeletal morbidity. A better understanding of macrophage‐driven inflammation in FL has resulted in targeted medical therapies such as Tocilizumab being utilized in FL patients. Since FL is a rare disease, minimal guidance on how treatment modalities should be utilized is available. We describe the case of a girl with FL presenting at 15 months with severe pain, swelling, and deformity of predominantly the small joints secondary to SCNs. Critical airway narrowing from laryngeal nodules necessitated tracheostomy. Regression of motor skills was also apparent. Fortnightly tocilizumab infusions improved pain and irritability, allowing neurological evaluation and tracheostomy decannulation. It did not halt the progression of SCNs. Therefore, we completed a 10/10 matched family donor haematopoietic stem cell transplant. Post‐transplant, she is stable neurologically, with resolution of her SCN, and her cognition, performance status, and well‐being are considerably improved by transplant.

1

**Key Points**

- This case series reported two cases of type 2/3 Farber's Lipogranulomatosis (FL) treated with Haematopoietic Stem Cell Transplant (HSCT).
- Case 1 was a 3y11m old girl who had a matched sibling transplant. She had a complicated transplant course including infections and pulmonary oedema. By day 450 post transplant her ESR had normalised and nodules had decreased significantly in size.
- Case 2 was a 3y10m old boy who had an unrelated donor transplant. By day 337 he had significant improvement of his subcuntaneous nodules and reduction of his ESR.
- This case series emphasizes that cases of type 2/3 FL can be treated with FL and provided useful information for us in our treatment decision making process.

## 2 Introduction

Farber's lipogranulomatosis (FL) is an autosomal recessive lipid storage disorder caused by deficient acid ceramidase activity. Seven different variants of FL have been described. Classical or Type 1 presents with the cardinal triad of arthritis, voice hoarseness, and SCN. These patients often have severe neurological complications and pass away by age 2–3. Types 2 and 3 are more mild versions of Type 1, and these often have a milder neurological phenotype. Types 4, 5, 6, and 7 also been reported but represent rarer subtypes of FL often reported in case reports or family pedigrees [1, 2]. The pathology of FL occurs secondary to inflammation resulting from accumulating pro‐inflammatory ceramides [1].

No clear guidance into optimal management strategies exists. For those who do not have CNS involvement, haematopoietic stem cell transplant (HSCT) can provide a viable option for improvement in morbidity and mortality. HSCT is theorized to work by two mechanisms: (a) providing acid ceramidase or (b) correcting leukocyte dysregulation as a result of ceramide accumulation [2].

Current literature of HSCT in patients with FL consists of case series and case reports [2, 3, 4, 5, 6, 7, 8, 9]. To the best of our knowledge, we could find 19 cases; these are summarized in Table 1. Age at transplant varies from 8 months to 21 years of age. The majority of cases have unique pathogenic variants causing disease, and currently no phenotypic‐genotypic correlation is reported in the literature [2].

**TABLE 1:** Table of all reported transplanted cases of Farber's lipogranulomatosis in literature and.

Year	Number of cases	Age at transplant	Mutation	Donor	Transplant outcome/follow‐up	References
2019 and 2006	10 7 Germany 2 Italy 1 Switzerland	1.3–21 years *One had 2 transplants*	7/10 available Heterzygous c.760 A>G in exon10, c.1175 G>A exon 11 Homozygote c.833 C>T Homozygote C917 + 5G>A Heterozygote c 410_411delAT, c.413A>T Heterozygote c.174_175insC, c.626G>A Heterozygote p.Asp331Asn and skipping of exon 6	3 MSD 2 MFD 5 MUD	*3–16 years* follow up for those alive 4/10 have passed away All had improvement in joint outcomes, not all retained mobility 3/10 had neurological sequelae following transplant	[2, 8]
2019	Case 1	8 montha	Homozygous c.458A > G	Cord	*28 months post‐transplant—Both passed away* Clinical delay in fine motor and gross motor prior to HSCT, one cherry red spot—Progressive corticosubcortical bilateral frontotemporal volume loss 1 year post HSCT—Improvement in arthritis and nodules—Passed away 37 months—respiratory failure	[5]
	Case 2	9 months	Homozygous c.458A > G	Cord	*7 years* post‐transplant Developmental delay and axonal hypotonia prior to transplant 15 months post‐transplant cerebral parenchymal atrophy Resolution of nodules (from conditioning therapy) Passes away 7 years post‐transplant from respiratory pneumonia	
2018	1	2 years 7 months	Homozygote c.830 C>A in exon 11	MFD	*24 months post‐transplant—Alive* Improved hoarseness of voice Improved mobility (walking)	[3]
2014	1	1 year 6 months	Heterozygous c.410‐411delAT, c.704G>A	MUD	*10 months—Alive* Decrease in nodules 2 months post Mixed chimerism 40% at 10 months	[9]
2013	1	9 years	Not stated	MUD Followed by Haploidentical	*8 years post‐transplant—Alive* Presented with spinal compression < 10% Chimerism sufficient to normalise acid ceramidase Transplant rejected 2 years post, rescued with haploidentical transplant Improvement in subcutaneous nodules, joint mobility and involved bone Has developed seizures post HSCT (initially absence then more generalized)	[6]
2004	Case 1	3 years 11 months	Not stated	MSD	*2.25 years* post‐transplant—Alive TRM: Grade II GVHD, Ciclosporin toxicity, Fracture Improved hoarseness of voice—Reduction in nodules (58 → 8), and improved mobility—89% chimerism	[4]
	Case 2	3 years 10 months	—	MUD	*1 year* post transplant TRM: Mucositis, Grade 1 GvHD, CMV reactivation Reduction in nodules (39 → 12), and improved mobility	—
2000	1	9.5 monhs	Not stated	MSD	*2.33 years* post‐transplant—*Passed away* Ceramidase activity in peripheral blood leukocytes increased from 6% to 44% 6 weeks post BMT Two months post patients SCN and hoarseness improved—loss of chimerism but ceramidase remained at heterozygous level—died from respiratory complications and patient had progressive neurological decline	[7]
1989	1	18 months	Not stated	Not stated	*6 months—Passed away* Neurological decline post‐transplant	

With the advent of personalized medicine, targeted medications have become available and open more options for patients with rare diseases such as FL. Interleukin‐6 is a cytokine that has been well described as important in the polarization and differentiation of macrophages in various disease settings [10, 11]. As a result of this, it has been hypothesized that Tocilizumab, an IL‐6 inhibiting monoclonal antibody, can improve symptoms in FL patients by reducing the inflammatory response [12].

We explore a case where both Tocilizumab and HSCT have been utilized in the management of a patient with FL.

### 2.1 Case

A 15‐month‐old girl was referred to tertiary pediatric rheumatology following the appearance of nodules on her fingers, toes, and ankles over the preceding 3 months. Hoarseness was also noted. At this time, there were no developmental concerns, and she had a full range of movement in her joints. She was referred to pediatric metabolic medicine at the age of 20 months following the detection of homozygous pathogenic variants in *ASAH1* (*c.998G>A, p.Arg333His*). Marked developmental regression, irritability, and pain had evolved since her first presentation. SCNs had increased in both size and distribution. Her dysphonia had worsened following an episode of croup, and biphasic stridor was evident at rest. Airway assessment under anesthetic revealed profuse laryngeal nodules and a pinpoint airway necessitating tracheostomy insertion. Due to the genetic diagnosis and clinical manifestations, we did not measure acid ceramidase activity pre‐transplant.

Pain management was ineffective using traditional analgesia, which limited meaningful assessment of her neurological status and development. Laboratory findings showed significantly elevated inflammatory markers (CRP: 30 mg/L, ESR: 104 mm/h). Fortnightly tocilizumab infusions were commenced, with subsequent improvements in pain and irritability. Increased use of her hands was noted. Inflammatory markers normalized within 3 months (CRP < 1 mg/L, ESR: 6 mm/h). Nerve conduction studies and a brain MRI were normal. Neuropsychological assessments were undertaken prior to tocilizumab. Pre‐treatment, her ability to engage with assessments was very poor. She demonstrated a Bayley‐III cognitive score in the extremely low range of ability. This improved to the low‐average range after treatment with tocilizumab, with significantly improved engagement, improved social skills, and receptive communication. After 12 months of tocilizumab, airway assessment was amenable to decannulation of the tracheostomy.

The evolution of new SCN's was, however, relentless and spread was noted to the larger joints, spine, plantar fascia, nasal cavity, and tongue. These contributed to a significant impact on her activities of daily living and development. She was subsequently referred for workup for HSCT. We completed a 10/10 matched family donor (MFD) transplant at the age of 3 years with fludarabine, busulfan, and alemtuzumab conditioning.

**FIGURE 1:**
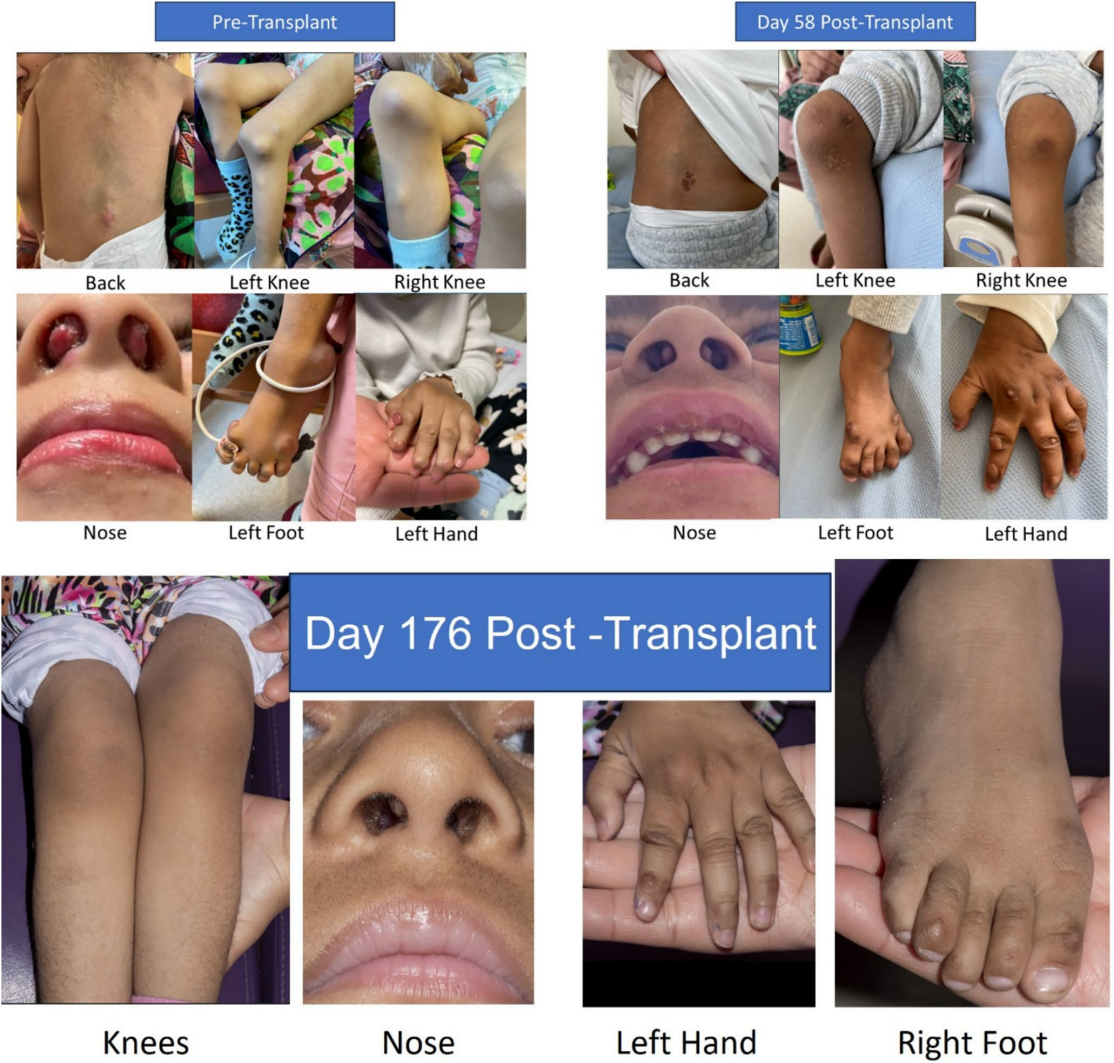
Clinical photographs of various affected anatomical regions. Photos taken pre‐transplant, day 56 post‐transplant, and day 176 post‐transplant.

During transplant complications included initial fever with alemtuzumab, mucositis requiring morphine infusion, stage 1 GVHD requiring steroids, and poor nutrition requiring total parenteral nutrition for 21 days. She was discharged on day 33. Chimerism at day 420 is 86.7% on peripheral blood, with 100% CD15 and 64.1% CD3 on short tandem repeat testing from peripheral blood. Acid ceramidase activity was slightly reduced on two separate occasions post‐transplant 1.47 nmol/kg/h (controls 2–2.3) and 1.09 nmol/h/ml (controls 1.9–2) but not deficient (CHU‐Toulouse laboratory). This may reflect the carrier status of the MFD. Her nodules over the post‐transplant period shrunk, developed black discoloration, and the majority have resolved by day 176 (Figure 1). She is neurologically stable 12 months post‐transplant. Neuropsychology assessment demonstrated intellectual abilities just below average ranges, with strengths in processing speed and working memory. Her Vineland‐3 report showed steady improvement in activities of daily living such that her social skills are now age appropriate. Motor and communication skills remain behind peers but are progressing well, and there have been no areas of regression.

## 3 Discussion

Farber's lipogranulomatosis (FL) is a rare diagnosis with no clear best practice management. We describe a case that has benefited from a multimodal approach utilizing tocilizumab and HSCT.

Current literature of management of FL patients focuses on HSCT or Tocilizumab and consists of case series and case reports [2, 3, 4, 5, 6, 7, 8, 9]. To the best of our knowledge, we could find 19 cases; these are summarized in Table 1. Age at transplant varies from 8 months to 21 years of age. The majority of cases have unique pathogenic variants causing disease, and currently no phenotypic‐genotypic correlation is reported in the literature [2].

Some clear themes emerge when reviewing HSCT in FL.

- Transplant resolves inflammatory joint granulomas and improves mobility.
- Patients with neurological involvement have a less favorable outcome and
- Matched family donors should be considered in donor selection for HSCT.

Transplant improves musculoskeletal arthritic manifestations of FL [2, 3, 4, 5, 6, 7, 8, 9]. Timeframes to resolution of arthritic symptoms and SCN's vary following transplant. One series reported resolution during conditioning therapy [5]. However, the majority report slow and steady improvement over the first two to 3 months post‐transplant [6, 7, 9]. Our case is in keeping with a slow and steady improvement, and this can be seen visually in Figure 1. In our case, tocilizumab improved symptom management but did not halt progression, which is in keeping with the one reported case series.

The literature demonstrates patients with neurological symptoms have more unfavorable outcomes [5]. Pre‐transplant neurological symptoms predict bad outcomes. The first two patients that received HSCT had neurological morbidity prior to and post‐HSCT; both had resolution of arthritis but marked neurological decline [7]. Two more cases reported in 2019 by a Canadian group also confirmed this finding. These patients aged 8 and 9 months went into transplant with delayed neurological milestones, and post‐HSCT both had neurological deterioration [5]. These cases most likely represent Type 1 FL, which doesn't respond as well to transplant as Type 2 or 3.

Matched family donors need to be considered as a donor source, as a number of patients have had successful HSCT from HLA matched family donors [2, 3]. Having mixed chimerism [6, 7] and transplanting using a carrier of a FL pathogenic gene does not impact HSCT outcome.

When reviewing Tocilizumab use in FL, a single multi‐center case series is reported. They describe five patients that had improved inflammatory markers (ESR) and improvement in pain and physical impairment following tocilizumab administration [12]. Our patient displayed similar improvements in inflammatory markers and pain but still had progression of SCN's leading to us progressing to HSCT for further management.

When assessing the best management strategy for a patient with FL, an accurate neurological assessment is required. In our patient, this is where the utilization of tocilizumab provided benefit, allowing for an accurate neuropsychological assessment with experienced neuropsychologists familiar with the challenges of children and young people with rare diseases, aiding transplant‐decision making.

In summary, we report a novel approach to managing a patient with FL utilizing tocilizumab to halt progression and allow accurate phenotypic assessment of FL type. This allowed HSCT to be completed with accurate knowledge of the patient's pre‐transplant neurological status.

## 4 Conclusion

FL patients with no primary‐CNS manifestations of FL benefit from HSCT. Accurate assessment of neurology is crucial to transplant decision making. This is often limited by presentation morbidity. Utilizing Tocilizumab to stabilize the disease process can aid in the transplant decision‐making process. Following HSCT, our patient had resolution in SCN, significant reduction in pain, and normalization of respiratory function by 176 days post‐transplant. Significant improvements in cognition and activities of daily living were also observed. Further work needs to clarify genotype–phenotype correlation in FL so we can differentiate early those that benefit from HSCT.

## Author Contributions

**Nathanael C. C. Lucas**, **Robert Wynn**, **Sarah Hulley**, **Chern Tan**, **Stewart Rust:** contributed to manuscript preparation. **Nathanael C. C. Lucas**, **Claire Horgan**, **Omima Mustafa**, **Srividhya Senthil**, **Denise Bonney**, **Ramya Nataraj**, **Robert Wynn:** contribute to the clinical HSCT management, review, and editing of the article. **Sophie Fisher**, **Chern Tan**, **Simon A. Jones**, **Sarah Hulley:** contributed to the metabolic management, review, and editing of the article. **Stewart Rust:** contributed to neuropsychological assessment, review, and editing of the article.

## Ethics Statement

The authors have nothing to report.

## Consent

The patient's family has consented to publication.

## Conflicts of Interest

The authors declare no conflicts of interest.

## Data Availability

The data that support the findings of this study are available on request from the corresponding author. The data are not publicly available due to privacy or ethical restrictions.
