# Correction to “Farber's Lipogranulomatosis: Multimodal Therapy With Tocilizumab and Consolidative HSCT Improves Assessment, and Long‐Term Outcome”

**DOI:** 10.1002/jmd2.70049

**Published:** 2025-11-10

N. C. C. Lucas, C. Horgan, O. Mustafa, et al., “Farber's Lipogranulomatosis: Multimodal Therapy With Tocilizumab and Consolidative HSCT Improves Assessment, and Long‐Term Outcome,” *JIMD Reports* 66, no. 5 (2025): e70041, https://doi.org/10.1002/jmd2.70041.

The key points are incorrect in the published article:

Key Points

- This case series reported two cases of type 2/3 Farber's Lipogranulomatosis (FL) treated with Haematopoietic Stem Cell Transplant (HSCT).
- Case 1 was a 3y11m old girl who had a matched sibling transplant. She had a complicated transplant course including infections and pulmonary oedema. By day 450 post transplant her ESR had normalised and nodules had decreased significantly in size.
- Case 2 was a 3y10m old boy who had an unrelated donor transplant. By day 337 he had significant improvement of his subcuntaneous nodules and reduction of his ESR.
- This case series emphasizes that cases of type 2/3 FL can be treated with FL and provided useful information for us in our treatment decision making process.

The correct key points are shown below:

Key Points

- We present a 15 month old who was diagnosed with Farber's Lipogranulomatosis (FL) without neurological involvement
- This case illustrates that a step‐wise approach to management of FL utilising Tocilizumab followed by haematopoietic stem cell transplant
- A stepwise approach allows assessment of neurological status and time to aid in transplant decisions in these complex patients.

We apologize for this error.

